# Inferior Vena Cava Calcified Thrombus Presenting With Abdominal Pain: A Case Report

**DOI:** 10.7759/cureus.5384

**Published:** 2019-08-13

**Authors:** Mohamed Ahmed, Rasha Saeed, May Abdulsalam, Samir Johna, Dina Elias

**Affiliations:** 1 Surgery, University of California, Riverside, USA; 2 Surgery, Arrowhead Regional Medical Center, Fontana, USA; 3 Family Practice, Ibn Albaldi Hospital, Baghdad, IRQ; 4 Surgery, Loma Linda University School of Medicine, California, USA; 5 Trauma, Riverside Community Hospital, Riverside, USA

**Keywords:** inferior vena cava, abdominal pain, calcified thrombus, pulmonary embolism

## Abstract

De novo thrombosis of the inferior vena cava (IVC) can cause significant morbidity and mortality. Calcified thrombus of IVC is an extremely rare incidental finding and is associated with recurrent deep venous thrombosis (DVT) and pulmonary embolism (PE). We present a case of abdominal pain secondary to a calcified thrombus in the supra-hepatic region of the IVC.

## Introduction

Calcified thrombus of the inferior vena cava is incidental imaging finding initially reported on CT scan of the abdomen in the pediatric population, which can resolve spontaneously with only a few reported cases in adults [1-3]. De novo inferior vena cava (IVC) thrombosis may be associated with hypercoagulable states, congenital or acquired pathology of the inferior vena cava [4]. We describe a case of calcified inferior vena cava thrombus in a 70-year-old female who presented to our emergency room with two months history of epigastric abdominal pain.

## Case presentation

**Figure 1:**
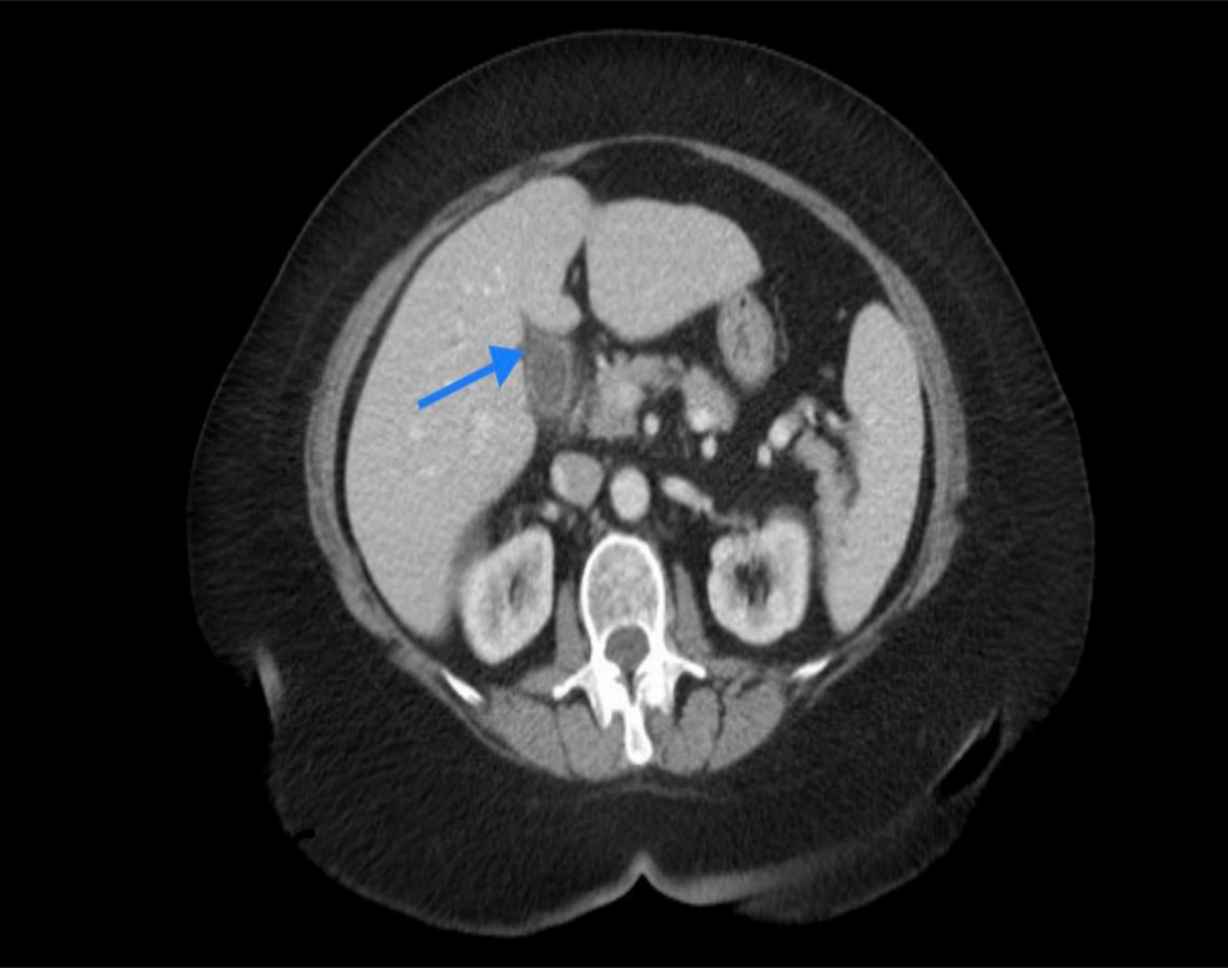
CT scan of the abdomen Acute emphysematous cholecystitis (blue arrow)

A 70-year-old female presented to our emergency room with two months history of "pounding, pinching" epigastric abdominal pain which did not improve with the use of prescribed proton pump inhibitors. Four days prior to her presentation the pain became more constant and had bilious vomiting. Relevant past medical history includes deep venous thrombosis with her pregnancy forty years earlier. Her vital signs were with in normal limits. Laboratory findings including liver function test were within normal limits except a white blood cell count 11.9 k/mm^3 ^(normal= 4.8-10.8), potassium 2.7 mmol/L (normal=3.5-5.1). Computed tomography scan of the abdomen and pelvis raised concern for emphysematous cholecystitis (Figure1).

**Figure 2:**
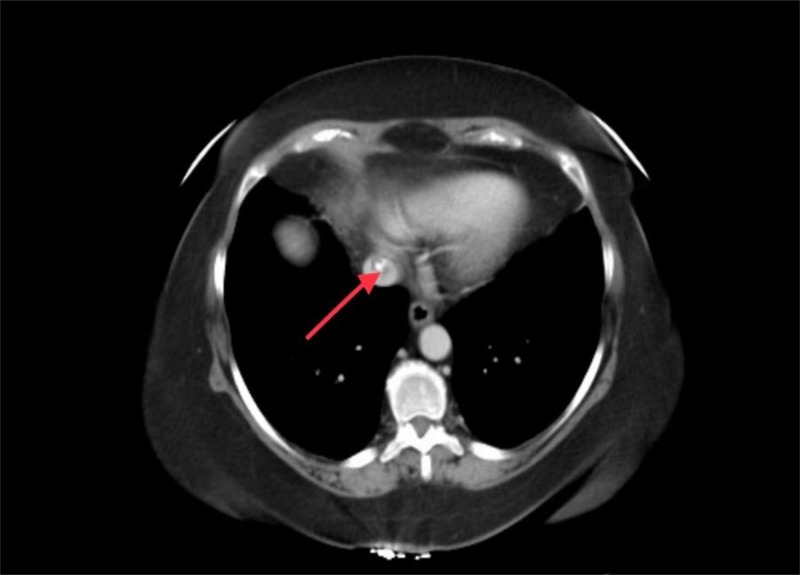
CT scan of the abdomen Calcified thrombus of the inferior vena cava (red arrow)

Results of the CT scan also raised the concerns for a calcified thrombus in the supra-hepatic inferior vena cava (Figure2).

Ultrasound of the abdomen revealed normal gallbladder wall thickness, patent portal vein, and inferior vena cava. Hepatobiliary iminodiacetic acid scan revealed delayed filling of the gall bladder consistent with chronic cholecystitis. An echocardiogram confirmed the diagnosis of calcified thrombus of the inferior vena cava. Anticoagulation in the form of heparin drip was administered. Hypercoagulable state workup revealed a low protein S activity of 53% (normal 63-140%). Patient symptoms dramatically improved the following day and she was discharged from the hospital two days after admission on an oral anticoagulant (apixaban).

## Discussion

Inferior vena cava calcified thrombus was first described as a post-mortem finding by Morgagni in 1769 and was first reported in 1961 by Singleton, but still continues to have unclear etiology [5]. This pathology was usually discovered incidentally and been referred to as "bullet thrombus" in multiple case reports introduced by Silverman [6, 7]. Virchow’s Triad (blood flow stasis, hypercoagulability, and endothelial injury) can explain the pathophysiology leading to IVC thrombosis. Thrombosis can occur in congenitally normal IVC as a result of compression from adjacent structures leading to stasis of the blood flow (renal cell tumor, pancreatic carcinoma, large uterine fibroid, Budd-Chiari syndrome), or secondary to hypercoagulable state and in cases of endothelial injury as with the use of IVC filter. One percent of the population have congenitally abnormal IVC (duplicate inferior vena cava, absence of infrarenal IVC, accessory left renal vein) resulting in turbulent blood that can lead to thrombosis in 60-80% of these patients [8]. Congenital atresia or hypoplasia of the IVC is a cause of recurrent deep venous thrombosis (DVT) in young adults [9]. Systemic embolization was reported only once in a child with transposition of the great vessels [10]. There are reported cases in association with adrenal hemorrhage in the neonate and with right atrial myxoma in a toddler [11, 12]. Calcified thrombus of the inferior vena cava can be the underlying pathology for recurrent DVT and pulmonary embolism (PE) [13]. Unexplainable low back pain with bilateral lower extremity deep venous thrombosis can be the presenting symptom [14]. Abdominal pain as a presenting symptom scarcely been reported and IVC thrombosis is not commonly thought of as the underlying cause. Useful diagnostic tests include Doppler ultrasound, echocardiogram, CT and MRI. A venogram is a sensitive test with the disadvantage of being invasive. The hypercoagulable state should always be ruled out and in our case, protein S deficiency proved to be the underlying pathology. The management of IVC thrombosis should be tailored to the underlying pathology which includes anticoagulation to prevent the propagation of thrombus, catheter-directive thrombolysis with the use of urokinase, tissue-type plasminogen activator (tPA), streptokinase (all carry a high risk for pulmonary embolism), thrombectomy (associated with a 2% mortality rate), caval interruption and endovascular stenting. While there are no general treatment guidelines, anticoagulation remains the mainstay of therapy and endovascular restoration of inferior vena cava patency is recommended when feasible [15, 16]. The American College of Chest Physicians recommends the use of oral activated factor ten inhibitors (rivaroxaban, apixaban, edoxaban) over vitamin K antagonist and low-molecular-weight heparin as a long term treatment of venous thromboembolic disease [17].

## Conclusions

Calcified inferior vena cava thrombus is an incidental finding associated with recurrent DVT/PE. In our case, epigastric abdominal pain was the presenting symptom and protein S deficiency was the underlying pathology. Long term anticoagulation with oral activated factor ten inhibitors (rivaroxaban, apixaban, edoxaban) remains the mainstay of therapy and restoration of IVC patency when feasible is recommended. 
